# Study of Efficacy of Extraosseous Local Infiltration of Multimodal Drug Cocktail for Pain Management After Total Joint Arthroplasty in Lower Limb

**DOI:** 10.7759/cureus.67483

**Published:** 2024-08-22

**Authors:** Ankur Salwan, Vivek H Jadawala, Aditya L Kekatpure, Salahuddin Ahmed, Geetika Malhotra, Yash C Parekh, Saksham Goyal

**Affiliations:** 1 Department of Orthopaedics and Traumatology, Jawaharlal Nehru Medical College, Datta Meghe Institute of Higher Education and Research, Wardha, IND; 2 Department of General Surgery, Jawaharlal Nehru Medical College, Datta Meghe Institute of Higher Education and Research, Wardha, IND

**Keywords:** periarticular, visual analogue score, pain management, epidural anesthesia, arthroplasty

## Abstract

Background

Osteoarthritis (OA) is a chronic debilitating condition involving joints that ultimately leads to chronic pain, restricted mobility, and functional impairment of the joint. The gold standard treatment of end-stage OA is arthroplasty. Following arthroplasty surgery, patients might have excruciating pain in the postoperative period. Our study aimed to evaluate the beneficial effects of a multimodal drug cocktail in patients who underwent arthroplasty surgery concerning postoperative pain and knee range of motion during the healing period.

Materials and methods

In this randomised case-control study, we enrolled 32 patients, of which 16 patients had knee arthritis and underwent total knee replacement. In contrast, the other 16 patients had hip arthritis and underwent total hip replacement. Randomisation was done using simple random sampling (envelope method), and patients were divided into groups A and B accordingly. Group A consists of a postoperative pain management modality using epidural analgesia with a top-up at a 12-hour interval. Group B consists of a postoperative pain management modality using an extraosseous multimodal drug cocktail consisting of clonidine, cefuroxime, tramadol, bupivacaine, adrenaline, and normal saline in specific quantities. Visual analogue scale (VAS) was assessed post-surgery for walking and resting. Joint range of movement and walking distance were assessed post-surgery and compared between the two groups.

Results

A total of 32 patients who had OA and underwent total knee arthroplasty (TKA) and total hip arthroplasty (THA) were selected and divided into case and control groups of eight, each by simple random sampling (envelope method). The mean preop VAS scores for the epidural and cocktail groups were 7.88 ± 0.61 and 7.44 ± 0.62, respectively, with p = 0.057, which is insignificant. However, when the groups were compared based on VAS score while standing at 24, 48, and 72 hours, the standing VAS score was found to be significantly higher among subjects, given epidural analgesia with p-values of 0.001, 0.001, and 0.001 at 24, 48, and 72 hours, respectively, which is significant in our study. Also, postoperatively, at 24, 48, and 72 hours, the mean degree of movement was found to be significantly higher among subjects, given cocktail analgesia with p-values of 0.013, 0.001, and 0.001, respectively.

Conclusion

As a result of early postoperative pain alleviation, the current study concludes that multimodal pain control procedures, which combine more than two medications with distinct mechanisms of action, successfully increase patient satisfaction. A multimodal medication conjunction administered locally to patients resulted in improved functional outcomes, faster recovery, and better rehabilitation.

## Introduction

Osteoarthritis (OA) is a prevalent joint condition that causes functional impairment and persistent pain. In addition to the degeneration and loss of articular cartilage, other symptoms include the development of osteophytes, ligamentous laxity, periarticular muscle weakness, and, in some instances, synovial cavity inflammation [1]. Even though disease progression is typically gradual, it can eventually result in painful joint failure [2]. According to a survey, about 3.3-3.6% of people worldwide suffer from OA [3]. Due to the population’s ageing trend, prevalence will rise soon [4]. Management is required to reduce discomfort and functional loss [5]. Total joint arthroplasty (TJA) of the hip and knee results in substantive and sustained improvement in quality of life for individuals with moderate to severe OA. It assists in reducing pain, restoring functionality, and also enhancing the quality of life associated with health. TJA, although showing successful results, is not without extended hospital stays and painful recovery. Extreme pain postoperatively has generally been associated with arthroplasty surgery, irrespective of the type that has been performed. Given this, it is critical to remember that each person’s sense of pain is unique and can be perceived broadly. About 60% of patients who have total knee arthroplasty (TKA) report severe postoperative pain, while about 30% of patients have moderate postoperative pain [6].

TJA may result in severe pain. Active physical therapy and rehabilitation are necessary to preserve joint range of motion but cannot be performed without the proper analgesics. This can delay hospital discharge and increase the risk of thromboembolism [7]. Despite all of these precautions, the length of recovery and rehabilitation depends significantly on pain management throughout the postoperative phase. Patient dissatisfaction brought on by inadequate pain management may result in subpar surgical results and a protracted recovery period. More than half of patients receiving TJA may endure unsatisfactory pain management and significant discomfort in the initial postoperative phase. Pain is still a complicated and poorly understood phenomenon.

Epidural analgesia or intravenous patient-controlled analgesia (PCA) has historically been used to manage pain after TJA surgery. The benefits and drawbacks of each strategy are, however, very different. Opioids, for instance, frequently cause drowsiness, disorientation, constipation, nausea, and vomiting, as well as pruritus, and they don’t always effectively relieve pain. While improved analgesia can be obtained with epidural infusions of local anaesthetics (with or without an opioid), they also carry a risk of hypotension, urinary retention, motor block that impairs ambulation, and spinal hematoma due to anticoagulation [8]. Periarticular analgesia and a multimodal pain control regimen have been developed and are becoming more widely accepted for the management of the aforementioned side effects.

Periarticular infiltration (PAI) is a newer, alternative localised analgesic known as local infiltration analgesia or periarticular multimodal drug injection (PMDI). It involves the injection of analgesic solutions into the adjacent tissues in the operative field. Local anaesthetics of amide derivatives, such as ropivacaine, bupivacaine, levobupivacaine, and others, as well as corticosteroids, opioids, epinephrine, and nonsteroidal anti-inflammatory medications, with dilution with normal saline, are typically used in this procedure. PAI using multimodal analgesia minimises the need for postoperative analgesia by reducing pain at central and peripheral levels [9]. It also blocks the pain influx at its origin and maximises muscle control, thus improving postoperative rehabilitation.

Regarding its effectiveness, several studies have confirmed the advantage of PAI in reducing postoperative pain, while other studies did not discover an improvement in pain control [10]. Thus, the current study is meant to test the effectiveness of extraosseous local infiltration of a heterogeneous drug cocktail against epidural anaesthesia in treating postoperative pain [11].

## Materials and methods

Methodology

The study has been approved by the Institutional Ethics Committee of Datta Meghe Institute of Higher Education and Research University with their letter number D.M.I.M.S. (D.U.)/I.E.C./2020-21/9377.

This study was undertaken at Acharya Vinoba Bhave Rural Hospital in Sawangi (Meghe), Wardha, India, on patients over the age of 18 who presented to the orthopaedics department for severe OA of both their knees and hips and were scheduled for arthroplasty surgery. The sample size for this study was 30, and of these 30 patients, two patients were operated on for bilateral OA knee and hip; hence, they were counted twice, making a total of 32. Inclusion criteria included adults above 21-80 years of age and advanced arthritis patients undergoing joint arthroplasty. Exclusion criteria included patients with spine deformities, patients with allergies to medications used in the multimodal cocktail, and patients with active infections.

A detailed history and clinical examination of all patients (previous surgery, drug reactions) was taken. Patients were explained about the study in detail. Written, informed, and verbal consent was obtained from the patients willing to participate in the study.

History of diabetes mellitus, prolonged medication, steroid intake, sickle cell, ankylosing spondylitis, alcoholism, hypertension, hemoglobinopathy, fractures, dislocations, and rheumatoid arthritis, if any were taken into consideration, deformities (adduction, abduction, and flexion), limb length discrepancies and preoperative pain using the visual analogue scale (VAS) for pain, analgesics used was noted. To avoid potential difficulties, all patients underwent a preoperative medical evaluation, which included clearance and fitness by a physician and an anaesthetist.

The patients were randomised into two groups by envelope randomisation. In the first group (group A), the patients received epidural analgesia for 72 hours postoperatively with pushes at 12-hour intervals. The epidural analgesia was given by using 0.5% bupivacaine. The preparation was done with the adjuvant being fentanyl (25 mcg). In the other group (group B), patients received an extraosseous multimodal drug cocktail injection intraoperatively. Standard protocol was followed for epidural and multimodal cocktail anaesthesia. The extraosseous local multimodal drug cocktail used for pain management includes the following (Table 1).

**Table 1:** Constituents of the multimodal drug cocktail mg, milligram; cc, cubic centimetre; µg, microgram

Medication	Dosage	Amount
Bupivacaine	0.5% (200-400 mg)	24cc
Clonidine	0.08 mg	0.8cc
Tramadol	50 mg	10cc
Cefuroxime	750 mg	10cc
Adrenaline	300 µg	0.3cc
Normal saline	0.9%	25cc

Local sites of infiltration for total hip arthroplasty (THA)

**Figure 1:**
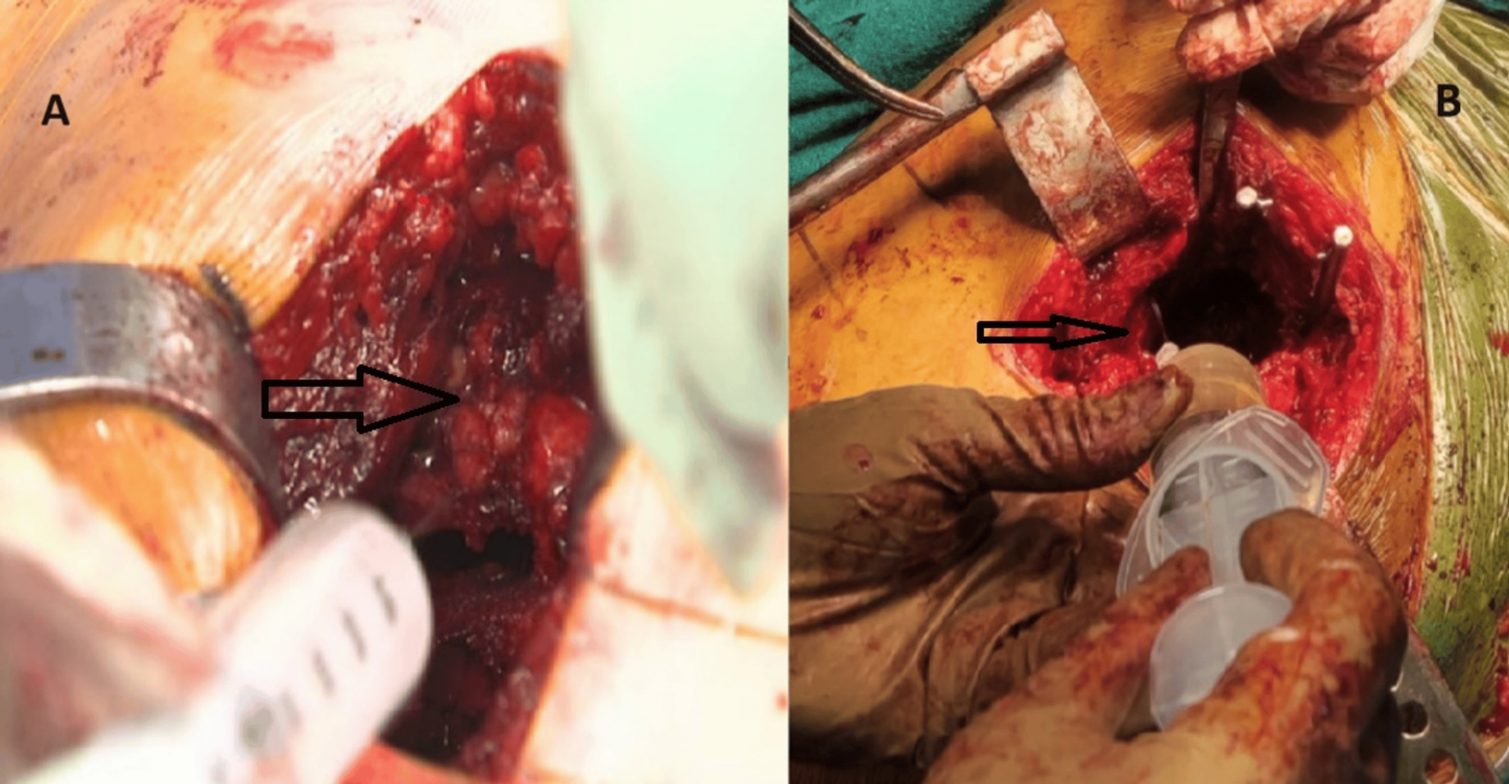
Local infiltration of the multimodal cocktail in the posterior capsule (A) and anterior capsule (B)

Before the final reduction, the cocktail was injected into the following structures: the posterior capsule, anterior capsule, iliopsoas tendon, and the insertion point (Figure 1).

**Figure 2:**
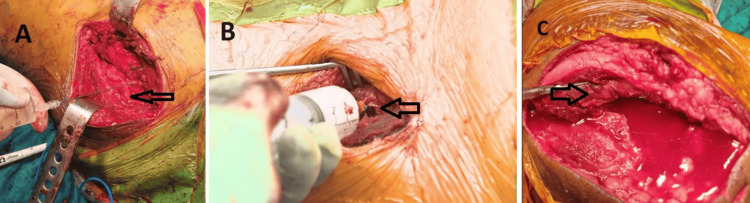
Local infiltration of the multimodal drug cocktail injection in the external rotators (A), tensor fascia lata (B), and gluteus maximus (C) of the hip joint

After the final reduction (after irrigation and before the final closure), the cocktail was injected into the following: abductors, short external rotators, fascia lata, gluteus maximus, and the insertion point (Figure 2).

Local sites of infiltration for TKA

**Figure 3:**
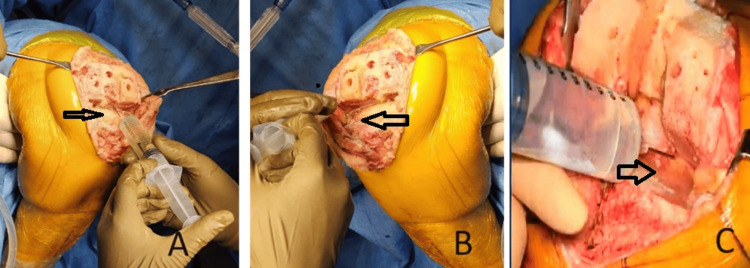
Local infiltration of the multimodal drug cocktail injection in the posterior capsule (A), lateral (B), and medial (C) menisci rim of the knee joint

Before liner insertion and reduction, the cocktail was injected into the following: posterior capsule, posteromedial, and posterolateral structures (Figure 3).

**Figure 4:**
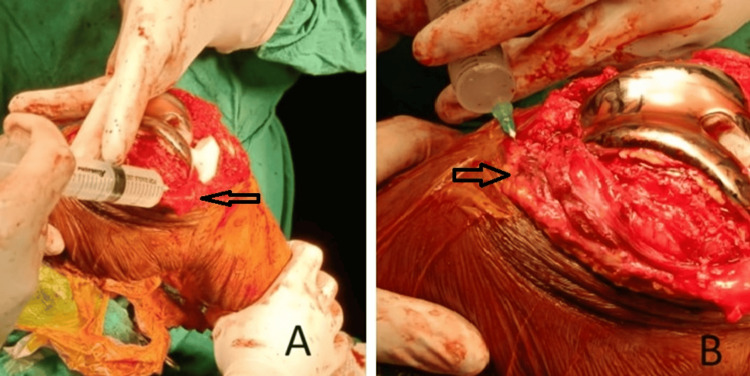
Local infiltration of the multimodal drug cocktail injection in the knee joint's infrapatellar fat pad (A) and extensor compartment (B)

After reduction, the cocktail was injected into the following: extensor (quadriceps) mechanism, synovium, pes anserinus, anteromedial capsule, periosteum, iliotibial band, collateral ligaments and origins, suprapatellar, and infrapatellar fat pads (Figure 4).

Statistical analyses

A Microsoft Excel spreadsheet was used to enter the data, and any discrepancies were reviewed. Tables and graphs were used to present the summarised data. The data was analysed using IBM SPSS Statistics, version 21.0 (IBM Corp., Armonk, NY). The variables that followed a normal distribution were determined using the Shapiro-Wilk test. Since the data had a normal distribution, parametric tests, such as the independent t-test, were used for bivariate analysis (for comparing two groups). The chi-square test was used for categorical variables. The level of statistical significance was set at a p-value less than 0.05.

## Results

Thirty-two patients were selected for the study and operated on for TJA. No significant differences between the two groups were found in demographic data (age and gender), as shown in Tables 2, 3.

**Table 2:** Mean age among study subjects

Variable	Modality of analgesia	N	Mean	Std. deviation	Std. error mean	p-value
Age	Epidural	16	47.88	17.254	4.314	0.669
	Cocktail	16	50.5	17.189	4.297	

**Table 3:** Gender distribution among study groups

Variables	Modality of analgesia				Total	
	Cocktail		Epidural			
	N	%	N	%	N	%
Gender						
Female	7	43.80%	10	62.50%	17	53.10%
Male	9	56.30%	6	37.50%	15	46.90%
Total	16	100.00%	16	100.00%	32	100.00%

Table 4 shows the intergroup comparison of walking and standing VAS at 24, 48, and 72 hours in hip patients. Mean value, standard deviation, and standard error were calculated. The p-value for the 24-hour walking VAS was 0.002, the 48-hour walking VAS was 0.001, and the 72-hour walking VAS was 0.019. The p-value for the 24-hour standing VAS score was 0.0005, the 48-hour standing VAS was 0.001, and the 72-hour standing VAS score was 0.006.

**Table 4:** Intergroup comparison of walking and standing VAS scores in hip patients VAS, visual analogue scale

Hip	Modality of analgesia	N	Mean	Std. deviation	Std. error mean	p-value
24-hour walking VAS score	Epidural	8	7.5	0.535	0.189	0.002
	Cocktail	8	6.5	0.535	0.189	
48-hour walking VAS score	Epidural	8	6.88	0.354	0.125	0.001
	Cocktail	8	5.75	0.707	0.25	
72-hour walking VAS score	Epidural	8	6.5	0.756	0.267	0.019
	Cocktail	8	5.5	0.756	0.267	
24-hour standing VAS score	Epidural	8	6.75	0.463	0.164	0.005
	Cocktail	8	5.75	0.707	0.25	
48-hour standing VAS score	Epidural	8	6.63	0.518	0.183	0.001
	Cocktail	8	5	0.535	0.189	
72-hour standing VAS score	Epidural	8	6	0.756	0.267	0.006
	Cocktail	8	4.88	0.641	0.227	

Table 5 shows the intergroup comparison of walking and standing VAS at 24, 48, and 72 hours in knee patients. Mean value, standard deviation, and standard error were calculated. The p-value for the 24-hour walking VAS was 0.002, the 48-hour walking VAS was 0.002, and the 72-hour walking VAS was 0.002. The p-value for the 24-hour standing VAS was 0.001, the 48-hour standing VAS was 0.001, and the 72-hour standing VAS was 0.001.

**Table 5:** Intergroup comparison of walking VAS and standing VAS scores in knee patients VAS, visual analogue scale

Knee	Modality of analgesia	N	Mean	Std. deviation	Std. error mean	p-value
24-hour walking VAS score	Epidural	8	8	0	0	0.002
	Cocktail	8	6.88	0.835	0.295	
48-hour walking VAS score	Epidural	8	7	0	0	0.002
	Cocktail	8	6.13	0.641	0.227	
72-hour walking VAS score	Epidural	8	7	0	0	0.001
	Cocktail	8	5.88	0.641	0.227	
24-hour standing VAS score	Epidural	8	7.38	0.518	0.183	0.001
	Cocktail	8	6	0.756	0.267	
48-hour standing VAS score	Epidural	8	7.25	0.463	0.164	0.001
	Cocktail	8	5.38	0.744	0.263	
72-hour standing VAS score	Epidural	8	6.38	0.518	0.183	0.001
	Cocktail	8	5	0.535	0.189	

Table 6 shows the intergroup comparison of the degree of movement and straight leg raise at 24, 48, and 72 hours in hip patients. Mean value, standard deviation, and standard error were calculated. The p-value for the 24-hour degree of movement was 0.080, the 48-hour degree of movement was 0.001, and the 72-hour degree of movement was 0.050. The p-value for the 24-hour straight leg raise was 0.001, the 48-hour straight leg raise was 0.005, and the 72-hour straight leg raise was 0.001.

**Table 6:** Intergroup comparison of the degree of movement and straight leg raise in hip patients

Hip	Modality of analgesia	N	Mean	Std. deviation	Std. error mean	p-value
24-hour degree of movement	Epidural	8	23.75	10.607	3.75	0.08
	Cocktail	8	33.75	10.607	3.75	
48-hour degree of movement	Epidural	8	36.25	8.763	3.098	0.001
	Cocktail	8	52.5	7.071	2.5	
72-hour degree of movement	Epidural	8	49.38	13.742	4.858	0.05
	Cocktail	8	63.75	13.025	4.605	
24-hour straight leg raise	Epidural	8	20	0	0	0.001
	Cocktail	8	30	5.345	1.89	
48-hour straight leg raise	Epidural	8	33.75	5.175	1.83	0.005
	Cocktail	8	46.25	9.161	3.239	
72-hour straight leg raise	Epidural	8	46.88	5.303	1.875	0.001
	Cocktail	8	68.75	7.906	2.795	

Table 7 shows the intergroup comparison of the degree of movement and straight leg raise at 24, 48, and 72 hours in knee patients, and they were found to be significantly more among subjects given cocktail analgesia.

**Table 7:** Intergroup comparison of the degree of movement and straight leg raise in knee patients

Knee	Modality of analgesia	N	Mean	Std. deviation	Std. error mean	p-value
24-hour degree of movement	Epidural	8	18.75	3.536	1.250	0.015
	Cocktail	8	25.00	5.345	1.890	
48-hour degree of movement	Epidural	8	29.38	1.768	0.625	0.001
	Cocktail	8	46.88	7.039	2.489	
72-hour degree of movement	Epidural	8	49.38	6.781	2.397	0.001
	Cocktail	8	66.25	9.161	3.239	
24-hour straight leg raise	Epidural	8	16.25	7.440	2.631	0.003
	Cocktail	8	30.00	7.559	2.673	
48-hour straight leg raise	Epidural	8	33.75	7.440	2.631	0.005
	Cocktail	8	46.25	7.440	2.631	
72-hour straight leg raise	Epidural	8	44.38	10.155	3.590	0.002
	Cocktail	8	65.00	11.952	4.226	

## Discussion

Painless THA or TKA can be accomplished using regional anaesthesia and multimodal pain management techniques that avoid unnecessary medicines [12]. After knee and hip arthroplasty, multimodal pain management has become the norm [13]. For patients having a TKA, a variety of preoperative, perioperative, and postoperative analgesia techniques have been documented. While there is evidence that epidural analgesia is beneficial, there are also known drawbacks, including the risk of spinal infection, neurogenic bladder, hypotension, respiratory depression, and pulmonary hypertension [14,15]. Periarticular injections have proven to be a valuable complement to multimodal pain management plans. In our study, delayed wound healing was present in three patients, of which two were in the periarticular group and one was in the epidural group. The incidence of nausea and vomiting was lesser among the periarticular group. Although there is wide variation in both the sites of injections and the components used in periarticular cocktails, there is minimal standardisation among surgeons’ injection methods.

It is appealing to offer local analgesia with less risk of systemic adverse effects in the vicinity of surgical trauma. Following knee surgery, intraarticular injections of various analgesics have been demonstrated to decrease the need for postoperative analgesia and may result in an earlier hospital discharge [16,17]. Multimodal analgesia for wound infiltration has long been a contentious topic. Several surgical procedures have described many techniques for administering local anaesthetics intraoperatively and postoperatively [18].

PAI using multimodal analgesia after TKA reportedly reduces postoperative analgesia requirements [19,20]. Additionally, by minimising problems such as respiratory depression, nausea, vomiting, ileus, urine retention, pruritus, hypotension, bradycardia, and cognitive abnormalities, the multimodal approach increases the safety of the procedure. These factors make a multimodal pain programme with periarticular injection significantly improving post-TKA and post-THA pain management [9].

There are 32 patients in our study, with patients divided into epidural and cocktail. In our study, the patients with left-side joint involvement were 46.9%, and the right-side joint involvement was 53.1%. No discernible changes were found between the two study groups when the side of the limb involved were compared. In our study of 30 patients, two patients who underwent bilateral joint arthroplasty and who, by coincidence, received an epidural for one side and a cocktail on the other side reported better pain relief in the operation where the modality of analgesia was a cocktail.

In 2009, Mullaji et al. evaluated the effectiveness of a periarticular injection of a mixture of opioids, corticosteroids, and local anaesthetic in patients undergoing bilateral TKR [21]. They delivered the drug mixture to one of the two knees. On the side that received the periarticular injection of the anaesthetic cocktail, as contrasted to the side that did not, they reported noticeably reduced pain levels and improved quadriceps recovery.

In our study, no significant differences were seen in the Pre-op VAS score among subjects given epidural or cocktail analgesia. Our analgesic cocktail consisted of bupivacaine, adrenaline, clonidine, tramadol, cefuroxime, and normal saline. Bupivacaine is a well-established long-acting local anaesthetic agent; when mixed with adrenaline, it helps in local vasoconstriction at the injection site, and it ultimately keeps bupivacaine localised to the area of injection, thereby prolonging the time of action of bupivacaine. Adrenaline also causes the contraction of smooth muscle fibres of arterioles and potentially minimises intraarticular bleeding. Clonidine, being an alpha antagonist, has an analgesic effect that is short-lasting (four hours after the injection), but when used in combination with opioid agents such as tramadol, it has a synergistic action as well as helps in reducing the dose of opioid dosage used for analgesia. We did not consider including steroids in the cocktail as previous studies reported no significant improvement in pain relief and early postoperative ROM. Steroids also pose a risk for surgical site infection. It is believed that TJA-related pain makes mobilisation and hospitalisation more difficult. Hence, with the utilisation of a periarticular multimodal drug cocktail, these inconvenient side effects could be countered.

In our study, the patients had significant pain relief clinically when compared to the epidural patients, as their ROM was found to be significantly more than the patients who received epidural analgesia and could walk a longer distance, which signifies the difference in pain relief in the clinical setting. We believe the changes in the VAS score in our study to be clinically significant, along with other measures of functional improvement and early rehabilitation.

Postoperative rehabilitation is essential to keep the postoperative risks of deep vein thrombosis and quadriceps weakening, limiting the range of movement of patients failing one of the primary aims of the arthroplasty surgery. Early rehabilitation also ensures a lesser hospital stay. The use of the analgesic cocktail was justified to promote contraction of the smooth muscles that line the arterioles, which could reduce intraarticular haemorrhage and lengthen the time the agents would operate locally. In this light, the cocktail’s epinephrine ingredient stands out remarkably. Jiang et al., in their study, revealed that following TKA, patients discovered that the PMDI group had a greater range of motion than the placebo group at 24, 48, and 72 hours after surgery; they also published the range of motion data for patients receiving THA, which similarly revealed that the PMDI group had a greater range of motion than the placebo group [22].

Limitations

The main limitation of our study was that the sample size was limited to only one centre. Secondly, since the study was done during an outbreak of the novel coronavirus (COVID-19), the number of patients who could be enrolled for elective TJA was limited. Thirdly, this study could not compare the outcomes between study groups with and without non-steroid anti-inflammatory drugs (NSAIDs). A similar study must be carried out with a larger sample size and in a community-based context to ensure that the results can be applied broadly. The functional outcome in the studies was analysed at 24, 48, and 72 hours; more extensive comparative studies assessing the long-term results would be preferable to validate the results.

## Conclusions

In conclusion, the present study shows that multimodal pain control protocols using a mixture of more than two drugs with different mechanisms of action effectively improve the patient’s rehabilitation by providing early postoperative pain relief without increasing the risk of complications in patients undergoing TJA. So, cocktail patients had more extended pain relief with early rehabilitation and functional outcomes. Periarticular multimodal analgesia injection may prove helpful in encouraging patients to undergo arthroplasty procedures.

## References

[1] Hutton CW (1989). Osteoarthritis: the cause not result of joint failure?. Ann Rheum Dis.

[2] Litwic A, Edwards MH, Dennison EM, Cooper C (2013). Epidemiology and burden of osteoarthritis. Br Med Bull.

[3] Bortoluzzi A, Furini F, Scirè CA (2018). Osteoarthritis and its management - epidemiology, nutritional aspects and environmental factors. Autoimmun Rev.

[4] Wood AM, Brock TM, Heil K, Holmes R, Weusten A (2013). A review on the management of hip and knee osteoarthritis. Int J Chronic Dis.

[5] Sen R, Hurley JA (2023). Osteoarthritis. StatPearls [Internet].

[6] Fu P, Wu Y, Wu H, Li X, Qian Q, Zhu Y (2009). Efficacy of intra-articular cocktail analgesic injection in total knee arthroplasty - a randomized controlled trial. Knee.

[7] Horlocker TT (2010). Pain management in total joint arthroplasty: a historical review. Orthopedics.

[8] Choi PT, Bhandari M, Scott J, Douketis J (2003). Epidural analgesia for pain relief following hip or knee replacement. Cochrane Database Syst Rev.

[9] Maheshwari AV, Blum YC, Shekhar L, Ranawat AS, Ranawat CS (2024). Multimodal pain management after total hip and knee arthroplasty at the Ranawat Orthopaedic Center. Clin Orthop Relat Res.

[10] Weber A, Fournier R, Riand N, Gamulin Z (2005). Duration of analgesia is similar when 15, 20, 25 and 30 mL of ropivacaine 0.5% are administered via a femoral catheter. Can J Anaesth.

[11] Pepper AM, Mercuri JJ, Behery OA, Vigdorchik JM (2018). Total hip and knee arthroplasty perioperative pain management: what should be in the cocktail. JBJS Rev.

[12] Ranawat AS, Ranawat CS (2007). Pain management and accelerated rehabilitation for total hip and total knee arthroplasty. J Arthroplasty.

[13] Ross JA, Greenwood AC, Sasser P 3rd, Jiranek WA (2017). Periarticular injections in knee and hip arthroplasty: where and what to inject. J Arthroplasty.

[14] Pettine KA, Wedel DJ, Cabanela ME, Weeks JL (1989). The use of epidural bupivacaine following total knee arthroplasty. Orthop Rev.

[15] Mahoney OM, Noble PC, Davidson J, Tullos HS (1990). The effect of continuous epidural analgesia on postoperative pain, rehabilitation, and duration of hospitalization in total knee arthroplasty. Clin Orthop Relat Res.

[16] Skinner HB (2004). Multimodal acute pain management. Am J Orthop (Belle Mead NJ).

[17] Strassels SA, Chen C, Carr DB (2002). Postoperative analgesia: economics, resource use, and patient satisfaction in an urban teaching hospital. Anesth Analg.

[18] Dahl JB, Møiniche S, Kehlet H (1994). Wound infiltration with local anaesthetics for postoperative pain relief. Acta Anaesthesiol Scand.

[19] Busch CA, Shore BJ, Bhandari R (2006). Efficacy of periarticular multimodal drug injection in total knee arthroplasty: a randomized trial. J Bone Joint Surg Am.

[20] Vendittoli PA, Makinen P, Drolet P, Lavigne M, Fallaha M, Guertin MC, Varin F (2006). A multimodal analgesia protocol for total knee arthroplasty: a randomized, controlled study. J Bone Joint Surg Am.

[21] Mullaji A, Kanna R, Shetty GM, Chavda V, Singh DP (2010). Efficacy of periarticular injection of bupivacaine, fentanyl, and methylprednisolone in total knee arthroplasty:a prospective, randomized trial. J Arthroplasty.

[22] Jiang J, Teng Y, Fan Z, Khan MS, Cui Z, Xia Y (2013). The efficacy of periarticular multimodal drug injection for postoperative pain management in total knee or hip arthroplasty. J Arthroplasty.

